# Cold Plasma-Induced Modulation of Protein and Lipid Macromolecules: A Review

**DOI:** 10.3390/ijms26041564

**Published:** 2025-02-13

**Authors:** Srutee Rout, Pradeep Kumar Panda, Pranjyan Dash, Prem Prakash Srivastav, Chien-Te Hsieh

**Affiliations:** 1 Department of Agricultural and Food Engineering, Indian Institute of Technology, Kharagpur, West Bengal 721302, India; sruteerout1997@gmail.com (S.R.); pps@agfe.iitkgp.ac.in (P.P.S.); 2 Department of Chemical Engineering and Materials Science, Yuan Ze University, Taoyuan 32003, Taiwan; 3 Department of Chemical Engineering and Biotechnology, National Taipei University of Technology (Taipei Tech), Taipei 10608, Taiwan; pranjyandash@gmail.com

**Keywords:** cold plasma, modification, amino acids, protein, lipid, polysaccharides

## Abstract

Nowadays, the food industry is prioritizing many innovative processing technologies that can produce minimally processed foods with superior and higher quality, lower costs, and faster operations. Among these advancements, cold plasma (CP) processing stands out for its remarkable capabilities in food preservation and extending the shelf life. Beyond its established role in microbial inactivation, CP has emerged as a transformative tool for modifying food biomolecules through reactive plasma species, addressing the versatile requirements of food industries for various applications. This review focuses on the interactions between reactive plasma species and essential food macromolecules, including proteins, lipids, and polysaccharides. The novelty lies in its detailed examination of how CP technology triggers structural, functional, and biochemical changes in proteins and lipids and explains the mechanisms involved. It connects fundamental molecular transformations to practical applications, such as enhanced protein functionality, lipid stabilization, and improved oxidative resistance. CP induces alterations in protein structure, especially in amino acid configurations, that can be applicable to the formulation of advanced gel, 3D printing, thermostable emulsions, enhanced solubility, and sensory materials. This review explores the ability of CP to modify protein allergenicity, its different effects on the mechanical and interfacial properties of proteins, and its role in the production of trans-fat-free oils. Despite its potential, a detailed understanding of the mechanism of CP’s interactions with food macromolecules is also discussed. Furthermore, this review addresses key challenges and outlines future research opportunities, positioning CP as a sustainable and adaptable approach for innovating next-generation food systems. Further research is crucial to fully understand the potential of CP for food processing, followed by product development.

## 1. Introduction

Cold plasma (CP) is an advanced non-thermal technology with wide applications across the food and pharmaceutical industries. CP has been emphasized particularly in the food industry, where it finds applications for the inactivation of enzymes, surface modification, microbial decontamination, and functional enhancement [1,2]. CP technology offers significant promise in agriculture, but several challenges need to be addressed for its effective adoption and sustained development. As the food industry increasingly adopts eco-friendly, non-thermal methods for modifying food macromolecules, a major challenge lies in deciphering the complex interactions between CP and various crops. CP also helps in the sanitization of food products, improves the germination rate of seeds, modifies food components, and inhibits enzymatic activities [3]. CP technology offers significant promise in agriculture, but several challenges must be addressed for its effective adoption and sustained development. CP generates minimal waste and thus is known as a “clean labeled technology”. It also aligns with different practices by amplifying physicochemical properties while reducing chemical dependency. Reactive oxygen species (ROS) and reactive nitrogen species (RNS) generated during secondary plasma processes are pivotal to its effectiveness [4]. Plasma can be categorized into high-temperature and low-temperature plasma based on the energy supply method and the amount of energy imparted to the plasma [5,6,7,8]. Recently, CP has found applications in different research areas, such as surface sterilization, enzyme inactivation, disinfection, and surface etching [9]. CP can function at atmospheric pressure, which makes this technology cost-effective and appropriate for real-world uses (seed sterilization, increasing seed germination and plant growth, and disease control in fields) [10]. However, non-thermal low-pressure plasma necessitates vacuum systems, permitting specific control over plasma parameters and creating high-purity plasma, which is ideal for surface treatments, biomedical applications (sterilization), and basic research [10]. In the food industry, CP acts as a critical tool for surface fumigation by enhancing polymer surface energy and sterilizing medical devices. Its applications span various food products, including meat, vegetables, dairy, eggs, poultry, fish, grains, spices, herbs, and sprouted seeds, underscoring its potential to ensure food safety and enhance quality.

CP has garnered significant attention in modifying proteins as a non-thermal, safe processing approach [11]. The most widely utilized technology in the food sector is DBD-CP. RNS, ROS, and high-speed particle etching are ways in which DBD-CP alters proteins more efficiently than other methods. This is because high-energy reactive species can induce the etching reaction to expose the protein surface’s active sites, increasing the affinity between the protein and water molecules. CP can alter the three-dimensional structure of proteins by breaking peptide bonds under highly reactive gases, such as nitrogen and oxygen. Peptide bond energies shift, causing protein unfolding due to these extremely potent oxygen radicals [12,13]. This phenomenon causes aggregations, polymerization, and disulfide formation, which can alter the structure and functionality of proteins. ROS can oxidize free amino acids and proteins, resulting in modifications such as the hydroxylation of aromatic rings and aliphatic amino acid side chains, the nitration of aromatic residues, the nitrosylation of sulfhydryl groups, the sulfoxidation of methionine, the chlorination of aromatic and primary amino groups, and the transformation of specific residues into carbonyl derivatives. Similarly, RNS primarily targets phenylalanine, tyrosine, cysteine, and methionine, causing nitration and oxidation. These oxidative processes can lead to polypeptide chain cleavage and the formation of cross-linked protein aggregates. Convenience, safety, and environmental friendliness are among the benefits of DBD-CP, which may process sample powder directly without pretreatment [14]. Soy protein isolate’s (SPI) water-holding capacity (WHC), solubility, emulsification, and foaming properties improved after CP treatment at 35 kV for 8 min [15]. Wang et al. [16] observed that the most effective method for enhancing the functional characteristics of chickpea protein isolate was CP treatment at pH = 12 for 30 s. Long-term CP treatment increased PPI’s stability and foaming ability [17].

CP treatment can substantially saturate double bonds in lipids, converting them into single bonds, as non-thermal technologies are mainly associated with lipid oxidation. This process amplifies oxidative stability and oil hydrogenation without catalysts or the formation of trans-fatty acids [18,19]. There has been no thorough review dedicated to exploring CP’s role in the hydrogenation of liquid oils. This review seeks to address that gap by analyzing the interactions between CP and food macromolecules, which highlights recent improvements and demonstrates how CP can offer a sustainable alternative to conventional methods for altering macromolecules.

## 2. Cold Plasma Principle and Its Technique

CP is an innovative, novel, solvent-free, and environmentally friendly technology with a broader range of industrial applications. Plasma is the fourth state of matter, discovered by Irving Langmuir in 1928, and arises from the complete or partial ionization of gases, forming a mixture of positively charged ions, neutral atoms, and free electrons, or molecules in various energy states [20]. This ionized state contains a wealth of reactive species, including positive and negative ions, ROS like OH, O_2−_, and O_3_, RNS such as NO, N_2_O, and NO_2_, ultraviolet radiation, and charged particles [21]. Plasma is broadly categorized into thermal and low-temperature types. Thermal plasma, which forms at high pressures (>10^5^ Pa) and requires significant energy (~50 MW), operates in thermal equilibrium but is unsuitable for food applications due to its high temperatures and energy demands. In contrast, low-temperature plasma includes quasi-equilibrium thermal plasma and non-thermal plasma, with the latter—commonly called CP—operating in a non-equilibrium state [22]. CP works at ambient temperatures, making it ideal for heat-sensitive materials. Here, the electrons are highly energetic, while heavier particles like atoms and ions remain more astonishing due to their limited momentum transfer [23]. CP is produced by passing an electric current through a gas under an applied voltage, triggering collisions that generate ions, radicals, and radiation across various wavelengths [24]. Electric discharge is the most common method for ionizing gases due to its efficiency. The ionization process, explained by Townsend’s theory, occurs as the voltage increases, propelling electrons to collide with gas molecules and creating avalanches of ions and electrons, which sustain the plasma [25]. All plasma is discharged by producing and multiplying electron avalanches during gas breakdown [26,27]. An avalanche of electrons and positive ions is created when these newly created electrons are accelerated in an electric field, ionizing and colliding with other atoms and molecules [28,29]. Paschen’s law further highlights the relationship between pressure and electrode spacing, facilitating CP generation at both atmospheric and reduced pressures [30].

## 3. Overview of CP Generation

### 3.1. Radiofrequency (RF)-Generated CP

**Figure 1:**
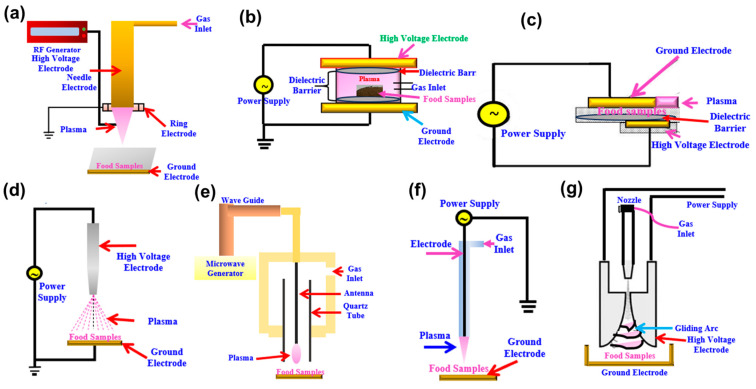
Schematic diagram showing generation of plasma by (**a**) radiofrequency, (**b**) dielectric barrier discharge, (**c**) surface DBD, (**d**) corona discharge, (**e**) microwave, (**f**) plasma jet, and (**g**) gliding arc discharge—adapted, redrawn, and modified from ref. [32] with copyright permission from Elsevier publication.

An RF power source operating at 13.56 MHz is commonly utilized to generate high-frequency plasma discharges (Figure 1a). This technique efficiently produces high-density plasma at low atmospheric pressure with minimal heat generation [31]. RF plasmas are categorized into three types: inductively coupled plasma (ICP), capacitively coupled plasma (CCP), and Helicon Wave (HW) plasma. Among these, ICP and CCP are widely used in industrial settings. As described by Rout et al. [32], CP can be generated either within a coil-shaped antenna or between two opposing electrodes. Interestingly, an electromagnetic wave has to deal with the penetration of a particular dielectric material. With this in mind, from Figure 1a, it can be observed that RF has more penetration depth and a longer wavelength than a microwave. This advantage ensures suitability for material heating with better uniformity. The disadvantages of RF technology are higher equipment and operational costs, lower power density, and slower heating rate, but it has high efficiency and output quality [33].

RF plasma sources have been effectively applied to modify starch and protein. This plasma treatment promoted the formation of amylose helices while eliminating amorphous starch regions, thereby enhancing thermal stability. Such innovations in starch modification have much potential for various applications, such as the formation of thermally stable semiconductors and food packaging materials [34].

### 3.2. Dielectric Barrier Discharge (DBD)

DBD plasma technology is gaining importance for its affordability, operational simplicity, straightforward construction, adaptability, and scalability. DBD (Figure 1b) offers flexibility in machine configurations and different options for dielectric material for its use in different applications [35]. A variation of dielectric barrier discharge (DBD) is surface dielectric barrier discharge (SDBD) (Figure 1c), which is generated by configuring electrodes in direct contact with a dielectric material. To expand the potential applications of SDBD, asymmetric electrode designs—where one electrode is insulated while the other remains exposed to air—have been developed, particularly for use in aerodynamic applications. In DBD, plasma is generated between two electrodes coated with dielectric materials like glass, silicon, ceramics, or plastic [36]. There is a gap of 0.1 mm to several centimeters between the electrodes, and these electrodes are insulated from different materials like ceramics, polymers, glass, or quartz. The dielectric layer prevents arc discharges and excessive machine heating for a stable and controlled environment. DBD technology generates various gases with rapid plasma and is exceptional compared to other types of CP as it operates without uniform discharge and gas flow [36]. These features make DBD plasma a practical and reliable tool for a broad spectrum of industrial, scientific, and research applications.

DBD is regarded as one of the safest plasma generation methods due to its ability to prevent spark and arc discharges by limiting current flow. However, it requires a high ignition voltage, often exceeding 10 kV, depending on the narrow electrode gap, necessitating specific precautions or insulation. Compared to microwave plasma, DBD generates a higher electron density and atomic oxygen concentration while minimizing temperature increases [37]. DBD plasma operates by producing microdischarges in the gas-filled space between two electrodes separated by a dielectric material. These microdischarges are short-lived, filamentary current channels formed by streamers—ionized gas regions propagating along the electric field lines. The microdischarges occur intermittently as the applied voltage alternates in polarity, creating a bright and uniform plasma glow. With low gas heating and high electron energy, these microdischarges drive various chemical reactions both within the gas phase and on the dielectric surface.

### 3.3. Corona Discharge (CD)

CD has a simple design and operates effectively at atmospheric pressure. It has pointed electrodes, basically named nail rows, which are mounted at one end of a hollow tube that extends outward to facilitate plasma generation [38]. A reactive zone is formed near the corona point through the ionization of plasma gas through the tiny pores of the tube (Figure 1d) [9]. This technology is cost-friendly, with low operational expenses and a simple setup operating in both direct current and pulsed voltage modes. In spite of its many advantages, CD has some drawbacks, like its limited processing area and uneven treatment application. To combat such problems and broaden CD’s application range, a multipoint-plate electrode configuration has been developed. This setup produces energized and focused plasma covering a larger area as compared to DBD systems [32,39]. These innovations emphasize the need to optimize electrode design, operational parameters, and materials to maximize the potential of CP technology in the food industry. CD plasma is used for surface decontamination, microbial decontamination, electro-precipitation, etc., but it is limited to non-homogeneous tiny areas [40].

### 3.4. Microwave (MW)-Based CP Generation

MW plasma generators (Figure 1e) produce electromagnetic waves, typically operating at a frequency of 2.45 GHz, to initiate plasma discharges. These waves interact with gas electrons, resulting in inelastic collisions, energy transfer, and ionization reactions [36]. MW plasma generation eliminates the need for electrodes and instead relies on the microwave electromagnetic field (MEF) for generating CP. This approach allows for precise control and facilitates the production of large-diameter plasma discharges, even under low-pressure conditions. MW energy is delivered to treatment chambers via waveguides, where it interacts with gas electrons to release energy for visible photons like ultraviolet (UV) through inelastic collisions [41]. MW plasma offers several benefits, including efficiently generating reactive species within ionized gases, making it particularly effective for sterilization and surface decontamination. Despite its promising capabilities, the broader adoption of MW plasma technology is constrained by its high operational costs and maintenance demands [42].

### 3.5. Plasma Jet (PJ)

PJ discharge (Figure 1f) involves various configurations, typically using an auxiliary plasma source to generate plasma [43]. The most prevalent design consists of electrodes arranged in a coaxial or two-ring configuration, allowing gas to flow through the system (Figure 1f). Radio waves, typically at a frequency of 13.56 MHz, excite the system, with the central electrode grounded while the outer electrode accelerates free electrons. These electrons collide with gas molecules, producing a wide array of reactive species [44]. A high-velocity gas, often a noble gas, carries the generated plasma beyond the electrode zone, delivering reactive species to the target material, such as a food product. This setup ensures a steady, homogeneous, and uniform discharge at ambient pressure, making PJ technology highly effective for various applications [42]. PJ offers the advantage of being directly applicable, and it can be effectively used in confined or small areas. While it is suitable for specific biological applications, but its use in food processing is limited due to the high costs associated with maintaining the required gas flow.

### 3.6. Gliding Arc Discharge (GAD)

The system comprises two identical electrodes positioned with a narrow gap at one end that progressively widens towards the opposite end [45]. A tube inserted into the narrower section supplies the working gas, and the flow rate is regulated using a gas flow meter (Figure 1g). When charged particles collide with the cathode, their temperature increases, promoting the thermal release of electrons [46]. The presence of a strong electric field facilities this emission. The effective operation of the device requires a high current flow of around 10 A and a voltage difference of approximately 100 V [47]. GAD plasma is categorized as warm plasma due to its moderate energy density and gas temperature, which fall between those of cold and thermal plasmas. While this technology shows potential for drying applications, research on its use and efficiency remains limited [3].

## 4. Different Factors That Affect CP Generation

The effectiveness of CP treatment is influenced by several power supply factors, including voltage, frequency, and exposure time [48,49]. Increasing the power input generally results in a higher electron density, which enhances the generation of reactive species and plasma activity [50]. Higher voltages, however, facilitate the dissociation of oxygen molecules into singlet oxygen atoms, leading to increased ozone production. The impact of these parameters in modifying food macromolecules varies depending on the product. For instance, Panngom et al. [51] observed that CP treatment at 50 W significantly improved the cooking properties and gelatinization of black glutinous rice compared to treatment at 40 W. Similarly, Miao et al. [52] reported that a voltage of 40 kV markedly enhanced the functional properties of myofibrillar proteins compared to other voltage levels. Additionally, Zhang et al. [53] and Amini et al. [54] demonstrated that the applied voltage and treatment time significantly influenced wheat flour’s functional and rheological properties, resulting in measurable improvements.

**Figure 2:**
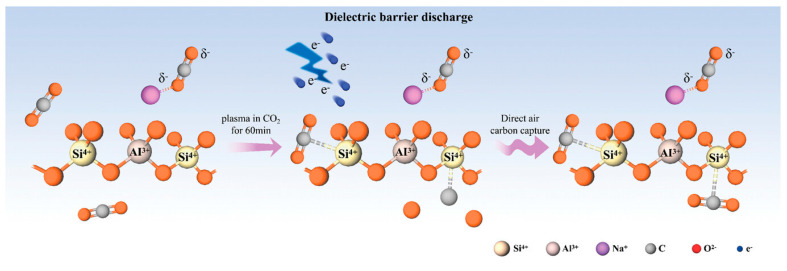
Schematic diagram modification of zeolite by the DBD in the CO_2_ atmosphere—adapted from ref. [55] with copyright permission from Wiley publication.

Relative humidity (RH) is another crucial factor influencing CP treatment efficacy. Shen et al. [55] compared different moisture conditions and found that the most significant inactivation effect during the moist treatments used was the DBD method, where CO_2_ was adsorbed within 60 min. This method successfully demonstrated efficiency and selectivity compared to the traditional method. The overall idea behind this study is provided in Figure 2, and CO_2_ does not affect any zeolite structure. In plasma discharge, water absorbs energy due to its molecular structure, quenching excited states, reducing electron energy and density, and ultimately decreasing plasma activity [56]. Water vapor can also reduce the surface resistance of dielectric barriers, leading to fewer microdischarges and less homogeneous discharges, which diminishes the production of reactive species [57]. However, for tapioca starch modification using DBD argon plasma at atmospheric pressure, Du et al. [58] observed higher cross-linking at a low RH (11%) compared to higher RH levels. Additionally, the RH can significantly alter the moisture content of the treated products, as shown by Zhang et al. [59], who found higher moisture in plasma-treated sodium caseinate films than untreated samples.

The choice of gas used in CP generation also affects the results, as the type of gas determines the ionization efficiency, the UV emission intensity, and the formation of reactive species [60,61]. Noble gases like helium and argon are commonly used due to their UV emission spectra and their high thermal conductivity (which aids in heat removal and lowering discharge voltages at atmospheric pressure) [62]. However, due to the short lifespans of reactive species generated by these gases (<10^−6^ s) and their high costs, air has emerged as a more practical and cost-effective alternative in recent research [63]. Additionally, the gas flow rate plays a critical role in CP treatment. It affects the speed at which reactive species reach the sample surface and influences the discharge operation, the residence time of reactive species, and mass transfer. Higher flow rates can accelerate the transportation of reactive species, enhancing treatment efficiency. However, if the flow rate is too high, the residence time becomes too short, and the excess active species may not be fully utilized, leading to decreased plasma activity [64]. Moreover, certain short-lived species may fail to reach the sample depending on the flow rate.

## 5. CP’s Effect on Different Proteins and Amino Acids

Proteins comprise diverse amino acids linked by peptide chains that define their structure and functionality [65]. A peptide bond connects the α-carboxyl group of one amino acid to the α-amino group of another by eliminating water, establishing the protein’s primary structure [66]. This structure determines the amino acid sequence, influencing the properties of the polypeptide chain. The secondary structure involves spatial arrangements such as alpha-helices, turns, beta-sheets, and random coils, shaped by hydrogen bonding, and peptide bond planarity [67]. A tertiary structure refers to the three-dimensional folding of α-helices and β-sheets into compact forms stabilized by interactions like hydrogen bonds, disulfide bridges, and ionic bonds [68]. Finally, the quaternary structure describes complexes formed by multiple polypeptide chains, stabilized through various bonds. The spatial arrangement of proteins is vital to their biological and functional properties [69]. In CP, the high-energy electrons generated play a crucial role in biomolecules such as amino acids, peptides, proteins, and DNA reactivity [70,71,72]. These electrons interact with surrounding gas molecules, forming reactive species such as free radicals, ions, and excited-state atoms [73,74]. Electrons in plasma trigger structural alterations in proteins by disrupting specific chemical bonds, altering side chain interactions and promoting cross-linking between protein molecules. When these reactive species come into contact with amino acids and peptides, they can induce modifications such as oxidation, deamination, and bond cleavage, altering proteins’ structural and functional properties. Electrons in CP facilitate oxidative modifications in amino acids such as tryptophan, tyrosine, and cysteine. CP treatment can alter protein structures, enhancing or modifying functionality through interactions between ROS, RNS, and amino acids. These changes depend on factors like the reactive gas, protein type, operating conditions, and amino acid composition [4]. Anuntagool et al. [75] identified excited atomic oxygen and nitride oxide in helium/oxygen plasma as contributors to protein activity loss, while Li et al. [76] emphasized oxygen’s role in hydrogen abstraction from protein backbones, leading to radical formation and chain cleavage. Reactive plasma species induce significant structural changes, including superoxide anion radicals, hydroxyl radicals, and nitric oxide. Roy et al. [77] demonstrated enzymatic activity loss, secondary structure unfolding, reduced tryptophan fluorescence, and increased molecular weight in lysozyme after exposure to these species. These effects arise from the chemical modifications of amino acid side chains, such as phenylalanine, cysteine, tryptophan, and tyrosine [78]. Similarly, oxygen radicals can decompose protein bonds (C-H, C-N, N-H), forming CO_2_, NO_2_, and H_2_O, while UV light below 250 nm disrupts amide bonds and secondary structures.

**Figure 3:**
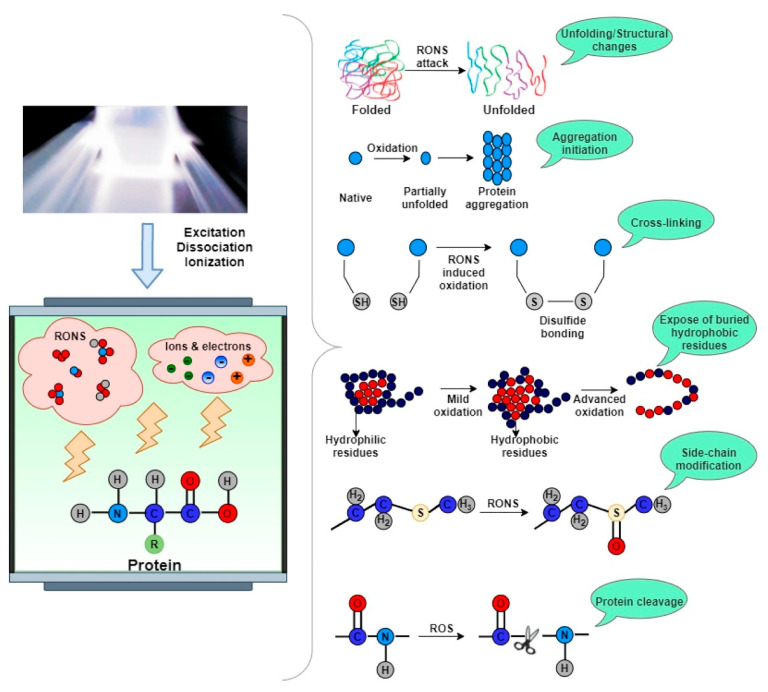
CP technique’s effect on proteins—adapted from ref. [80] with copyright permission from Elsevier publication.

ROS and RNS have distinct impacts on proteins. ROS cause amino acid oxidation, aromatic group hydroxylation, and carbonyl derivative formation, while RNS lead to the nitration and oxidation of phenylalanine, tyrosine, cysteine, and methionine [79]. Kopuk et al. noted that sulfur-containing and aromatic amino acids are particularly susceptible to plasma-induced modifications, such as oxidation, sulfonation, hydroxylation, and peptide bond cleavage, resulting in cross-linking and unsaturated bond formation (Figure 3) [80]. The spatial structure of proteins is closely linked to their functional and biological properties. CP treatment can modify this structure and, consequently, the functional properties of proteins. These changes result from interactions between chemically reactive plasma species and amino acids, causing structural alterations. The extent and nature of these modifications depend on factors such as protein type, the reactive gas used, the source of plasma, operating parameters, sample size, and the specific amino acid composition. Rout and Srivastav [32] investigated the effects of direct argon plasma exposure and UV-VUV photon emissions on l-alanine, observing significant degradation of COOH and CNH_2_ groups, with VUV photons demonstrating greater efficiency. Surface etching by ROS was also more impactful than chemical degradation from ion bombardment or radical irradiation. Laika et al. explored the interaction between DBD CP and Lactate Dehydrogenase (LDH), reporting secondary structure changes such as reduced alpha-helices, increased β-sheet content, and peptide polymerization [81]. The oxidation of specific amino acids, like histidine-195 at LDH’s active site, led to enzymatic activity loss.

Overall, CP treatments induce various modifications in amino acids, peptides, and proteins, altering secondary and tertiary structures, carbonyl and sulfhydryl content, hydrophobic residue exposure, and cross-link formation. Khan et al. evaluated DBD plasma treatment (air plasma at 60–70 kV for 5–10 min) applied to wheat flours, noting improved rheological properties, enhanced disulfide bonding, and secondary structure modifications [82]. FTIR analysis revealed structural disorder in strong wheat flour and increased order in weak wheat flour, with changes depending on the processing parameters and flour type.

### 5.1. Plant Proteins

Cereals and pulses are essential components of the human diet, providing starch and protein as key nutrients, alongside bioactive compounds, essential fatty acids, and dietary fiber [83]. Cereals belonging to the *Graminaceae* family include staples such as rice, maize, wheat, oats, barley, sorghum, rye, minor millets, and pearl millet [84]. Their composition typically comprises 70–72% carbohydrates, 7–15% protein, and 1–12% fats. Seed storage proteins, predominantly globulins, glutelins, and prolamins, are critical for nourishing developing embryonic tissues. Wheat gluten, a cereal protein, is particularly valued in baking, where its unique properties form a rigid structure to stabilize gelatinized starch during proofing and baking.

Pulses, such as peas, chickpeas, lentils, and beans, offer 17–30% protein and 4–23% carbohydrates, making them a sustainable dietary complement to cereals [85]. They are also rich in vitamins, dietary fiber, and minerals. Pulse proteins are available in various forms: flours (less than 65% protein), concentrates (over 65% protein), and isolates (over 90% protein on a dry weight basis). These proteins exhibit functional properties such as solubility, emulsification, foaming, thickening, water binding, fat binding, gelation, and flavor binding, which depend on factors like chain length, branching patterns, molecular weight, and hydrophobicity.

CP treatment has emerged as a promising method to enhance the techno-functional properties of proteins derived from cereals and pulses, facilitating their large-scale application [86]. For instance, the ACP treatment of SPI at 80 Hz for 1–10 min has increased protein solubility by unfolding and exposing hydrophobic groups. Mehr and Koocheki investigated the effects of DBD CP treatment on grass pea protein isolate at 9.4 and 18.6 kVpp, with exposure durations of 30 and 60 s and an oscillation frequency of 20 kHz. By employing optical emission spectroscopy to analyze reactive species, they observed that treatment at 18.6 kVpp for 60 s significantly improved creaming stability [87]. This enhancement was attributed to particle size reduction due to etching, leading to improved thermodynamic stability and increased surface hydrophobicity through enhanced electrostatic repulsion. Additionally, lower interfacial tension values suggested a more organized arrangement of secondary and tertiary protein structures, with proteins forming nanoparticles at the oil–water interface. The effect on the process parameters of CP on plant proteins is given in Table 1.

### 5.2. Dairy Proteins

Dairy milk, primarily derived from bovine species, is widely consumed and exported in both processed and fresh forms [88]. Recently, milk from goats and camels, as well as plant-based alternatives, has gained attention. Bovine milk consists mainly of water, with proteins, fats, minerals, and salts making up the remaining composition. The protein content in whole milk ranges from 32 to 38 g/L, with caseins and whey making up most of the protein (approximately 80% and 20%, respectively). Casein is recognized for forming stable aggregates through interactions with calcium and phosphorus, thereby maintaining the stability of these elements in milk. Due to the presence of large propyl residues, casein lacks a well-organized secondary structure and typically exists in micellar complexes, where α and β-caseins exist in the core, and κ-caseins stabilize the complex’s surface, preventing flocculation and maintaining stability [89]. Whey proteins, including α-lactalbumin, serum albumin, β-lactoglobulin, and immunoglobulins, are non-phosphorylated, globular proteins with a significant number of disulfide bonds, and they have a more structured secondary structure, including an α-helix, β-pleated sheets, and β-turns. Because of these stable structures, whey proteins are more susceptible to denaturation under heat or harsh environmental conditions.

Traditional milk processing involves heat treatments such as pasteurization at temperatures of 62.8 °C or higher for at least 30 min or 71.7 °C for at least 15 s. These methods are effective at reducing microbial contamination and improving milk safety but can also cause protein denaturation, off-flavors, a loss of essential vitamins, and non-enzymatic browning. Excessive heat increases viscosity and deteriorates protein solubility, resulting in aggregation, all of which affect the milk’s quality and shelf life [90]. Consequently, researchers have been using different non-thermal methods (CP, pulsed light) to inactivate microorganisms and enzymes as compared to traditional thermal treatments.

CP has been known to alter the properties of proteins in milk, including changes in color, viscosity, nutrient content, flavor, and particle size [91]. The viscosity of food products increases with the prolonged treatment of macromolecules like proteins, lactose, and fats. For instance, a study by Manoharan et al. showed no significant change in protein content, with values of 35.3 g/L ± 0.06 in treated milk versus 34.7 g/L ± 0.17 in the control sample [92]. However, Ng et al. found that exposure to plasma for 30 min reduced antigenic proteins, including α-lactalbumin and casein, due to cross-linking, protein aggregation, and fragmentation. These changes significantly reduced protein solubility [93]. CP-generated radicals are known to promote cross-linking, strengthening polymer networks, which have applications in developing barrier packaging films. Segat et al. [94] studied the impact of CP on whey protein isolate (WPI), observing a decrease in pH that affected solubility and increased the carbonyl content, emulsion stability, and foaming behavior. While the foaming capacity decreased with extended exposure (over 15 min), the foam stability increased [94]. These changes in hydrophobicity contribute to improved emulsion and foaming properties.

### 5.3. Meat and Aquatic Proteins

Meat-derived proteins are classified into three main types: sarcoplasmic proteins, myofibrillar proteins, and stromal proteins [95]. These proteins are valued for their functional properties, including heat-induced aggregation, hydration, gelation, WHC, and emulsifying ability. Myofibrillar proteins constitute 55–60% of total meat proteins and are particularly crucial for determining meat texture, yield, and flavor [96]. Their superior gel-forming and binding abilities make them essential in comminuted and emulsified meat products. However, their sulfur-containing amino acids, such as methionine and cysteine, are prone to oxidative damage. Research by Takai et al. highlighted methionine’s high susceptibility to oxidation, along with other reactive amino acids like tyrosine, phenylalanine, and tryptophan [97].

The functionality of meat proteins is influenced by intrinsic factors (e.g., body pH, stunning method, rigor state, amino acid composition, and protein structure) and processing conditions (e.g., pH, metal salts, temperature, additives, and processing methods). Thermal processing can lead to protein denaturation, coagulation, cross-linking, and aggregation, which reduce solubility and water-holding capacity [98]. These changes also alter flavor and color through Maillard reactions. While traditional methods to enhance the myofibrillar protein matrix involve additives and processing adjustments, emerging non-thermal technologies like CP treatment offer an eco-friendly alternative to enhance functionality with reduced reliance on additives [99].

A study by Pérez-Andrés et al. investigated the effects of atmospheric CP treatment (80 kV for 15 min) on hemoglobin, bovine lung protein, and pork gelatin [100]. Overexposure to plasma caused protein unfolding, which increased hydrophilic chains at the expense of hydrophobic regions, resulting in reduced emulsion stability. However, partial denaturation improved the WHC of pork gelatin and lung protein isolates, suggesting potential applications in fabricated foods requiring enhanced gelling properties.

The study also observed that the proteolytic activity was highest at an acidic pH, while the CP treatment reduced the activity of crude protease extracts [101]. Factors influencing enzyme inactivation included voltage, exposure time, enzyme structure, and the buffer environment. The acidification of the food matrix was attributed to the formation of HNO_2_, HNO_3_, and hydronium ions [102]. At higher treatment intensities, the emulsifying behavior further declined due to protein aggregation.

CP treatment also enhanced the textural properties of protein gels, such as their hardness, adhesiveness, and cohesiveness, by promoting cross-linking reactions through reactive species. The aroma-binding properties of myofibrillar proteins in dry-cured bacon showed increased aldehyde-binding capacity [103]. This was linked to hydrophobic amino acids and extended chain lengths resulting from secondary structure unfolding. The interaction between flavor compounds and myofibrillar proteins was governed by intermolecular forces such as disulfide bonds, hydrogen bonds, van der Waals forces, and ionic interactions [53]. These protein–flavor interactions can be reversible or irreversible, depending on the amino acid composition and the nature of the intermolecular forces.

**Table 1:** Effect of the process parameters of CP on plant proteins.

Plant Protein	Cold Plasma Type	Process Parameters	Key Findings	References
Chickpeas	APPJ	30 s	pH-shifting and CP combined modification were effective methods for improving the functional properties of protein isolates.	[16]
Sunflower Seeds	Dielectric Blocking Device	50 W for 0, 1, 2, 3, 4 and 5 min	CP had a significant effect on the characteristics of sunflower seed proteins.	[104]
Pea Protein Isolate	DBD	25, 30 and 35 kV for 2, 4, 6 and 8 min	Modified PPI can be more effectively used as thickening agents for different product formulations.	[32]
Soy Protein Isolate	DBD	25, 30 and 35 kV for 2, 4, 6 and 8 min	There was an increase in β-turn and β-sheets and a decrease in α-helix after the CP treatment.	[32]
Mung Bean	ACP	80 kV and 230 V for 10 min at 50 Hz	The gelling concentration of CP-treated beans was reduced to 14%. The firmest gels were formed by plasma.	[105]
Grass Pea	DBD	9.4 and 18.6 kVpp (5 and 10 min)	L-CPT increased aggregation and intermolecular β-sheets.	[87]
Oat protein	ACP	170 V–230 V for 15 and 30 min	CP modified foaming, functional, rheological, morphological, and characteristics	[106]
Brown rice	CPP	400 W for 5 min at 130 Pa	The phytic acid content was reduced after CP treatment.	[31]
Pea Protein concentrate	ACP	0–30 kV for 10 min at 3500 Hz and 0–1 A	The 10–20 min plasma-treated pea suspension (14 wt%) heated at 70 °C resulted in good mechanical gels.	[107]
Quinoa	DBD	50–60 kV at 37.2 kHz for 5–10 min	CP treatment substantially changed protein and starch structure	[108]
Almond	PJ	17 V and 2.26 A for 5–20 min	Plasma had no effect on peroxide value, color, and sensory attributes. The 10 min treatment sample was accepted.	[109]
Flaxseed	APPJ	2 min at pH 9	Plasma caused a decline in zeta potential values and pH and increased particle size during 0–120 s treatment	[110]
Zein	ACP	40, 50, 60 V for at 1.5 A for 2 min	ACP-treated samples showed higher encapsulation efficiency, solubility, and stability	[111]

APPJ: atmospheric-pressure plasma jet, ACP: atmospheric cold plasma, L-CPT: long–cold plasma treatment, DBD: dielectric barrier discharge, CPP: cold plasma pretreatment; PJ: plasma jet, PPI: pea protein isolate.

## 6. CP’s Effect on Lipids

CP, an advanced oxidation technique, has been widely explored for its impact on lipid oxidation, particularly in foods high in unsaturated fatty acids. Lipids with multiple double bonds, such as linoleic acid and linolenic acid, are especially prone to attack by reactive species due to the low energy required to abstract hydrogen atoms from C-H bonds near double bonds [112]. The oxidative effects of CP are predominantly driven by oxygenated reactive species, including atomic and singlet oxygen. CP also induces lipid oxidation via the Criegee mechanism, aldehydes, and hydroperoxides, forming ozonides and carboxylic acids.

Lipid oxidation increases with an increase in plasma power, treatment durations, and storage time, as these factors increase the levels of ROS and RNS [113]. However, studies have reported that CP treatment does not significantly induce lipid oxidation under certain conditions. For instance, investigations on foods like chicken breast [114], beef loin [115], canned ground ham [116], and meat batter [117] reported negligible lipid oxidation. This suggests that lipid oxidation depends primarily on the processing parameters (e.g., plasma power, gas type, exposure duration), intrinsic sample characteristics (e.g., fat content and composition), and handling conditions before and after treatment. The presence of antioxidants and the application of appropriate precautions can minimize or delay CP-induced lipid oxidation [82]. These measures control the oxidation of lipids and other undesirable changes, making CP a promising tool for the food industry without affecting lipid quality.

CP is a sustainable, low-temperature, atmospheric-pressure method for producing partially hydrogenated oils [118]. Unlike conventional hydrogenation, CP eliminates the need for high temperatures, high pressures, or catalysts such as nickel, thereby preventing the formation of harmful trans-fatty acids. Traditional hydrogenation methods, which add hydrogen to unsaturated fatty acids to convert oils into semi-solid or solid forms, improve oxidation and flavor stability but often produce trans-fats due to extreme processing conditions. In contrast, CP generates atomic hydrogen within the plasma, selectively saturating double bonds at low temperatures, avoiding the formation of trans-fatty acids [119].

In a study, Kopuk et al. [80] applied DBD plasma at 90 kV for up to 12 h using gas mixtures of 100% hydrogen, 5% hydrogen, and 95% nitrogen. This treatment reduced the iodine value (from 133 to 92) and unsaturated fatty acid content by 16.2% while increasing saturated fatty acids by 12%. These changes altered the oil’s physical properties, including a higher viscosity and the development of non-Newtonian behavior after 12 h of treatment. A subsequent study by Yepez et al. [18] found that CP treatment could produce oil fractions in liquid, gel, or solid states depending on the treatment duration. This process significantly decreased polyunsaturated fatty acids while increasing saturated fatty acids in soybean oil. Similarly, Puprasit et al. [19] investigated the CP hydrogenation of vegetable oils using a helium–hydrogen gas mixture (5–25% H_2_) at 31–100 °C for 1–20 h. Palm oil, with its higher natural saturation, achieved hydrogenation more efficiently than soybean oil. Optimal results were achieved with 15% H_2_ at an initial temperature of 31 °C (rising to 50 °C) and with an 8 h treatment duration, yielding iodine values comparable to commercial margarine. Remarkably, this process generated minimal trans-fats (1.44% after 4 h), with a trans-fat formation rate 6.12 times lower than that of traditional methods. Hydrogenation by CP has certain advantages, such as eliminating catalysts and trans-fat-free processing, but the high cost of working gases is a great challenge. This limitation could be overcome by using closed systems with gas recirculation, as reported in [120], making CP a promising alternative for the food industry for the hydrogenation of oil and lipids.

## 7. Combination of CP with Green and Emerging Technologies

The application of CP technology alongside other innovative techniques has been widely explored to enhance microbial inactivation efficiency. Integrating CP with other advanced food processing methods, whether applied concurrently or as pre- or post-treatment, has demonstrated significant potential in improving microbial elimination. For instance, Namjoo et al. investigated the combined effects of cold plasma treatment, ultrasound, and air drying on the quality of cumin seeds, showcasing the synergy between these approaches [121]. The study specifically examined water diffusivity, color changes, and microstructural modifications. To achieve this, cumin seeds were placed in polyethylene (PE) bags and exposed to nitrogen plasma treatment for 15 s. This was followed by ultrasound treatment at 180 W and subsequent air drying. The low-temperature plasma pretreatment significantly increased moisture diffusion rates, reducing the drying time and energy consumption while minimizing color changes during the ultrasonic air-drying process. Additionally, combining CP with pulsed electric fields (PEFs) has proven highly effective in microbial reduction. For example, *Litopenaeus vannamei* shrimp were first pre-treated with PEF, then immersed in an extract from Chamuang leaves, and subsequently exposed to high-voltage cold atmospheric plasma. Finally, the shrimp were packaged in a modified atmosphere, enhancing their storage stability over an extended period [122]. Implementing these measures efficiently inhibits the growth and spread of microbes in *Litopenaeus vannamei*, significantly increasing its storage lifespan.

The combined use of CP technology offers significant benefits for microbial decontamination and the disinfection of packaging surfaces, effectively minimizing the risk of contamination during storage and transportation. CP is a highly adaptable technology that can be applied to a diverse range of food products, such as fruits, vegetables, meat, processed foods, and packaged meals, at different stages of production [123]. This versatility makes it suitable for both small-scale and industrial food processing. Additionally, when CP is integrated with other preservation techniques, it can produce synergistic effects that enhance food safety and extend the shelf life of food products [39].

## 8. Future Prospects of CP

CP technology holds great potential for applications in agriculture and the food industry, especially in microbial decontamination and hazard mitigation. Although substantial advancements have been made in research, this innovative method is still in the early stages of development. As the food industry increasingly adopts eco-friendly, non-thermal methods for modifying food macromolecules, a major challenge lies in deciphering the complex interactions between CP and various crops. Optimizing conditions for different plant species and environmental factors is critical to maximizing the benefits of CP technology while minimizing potential adverse effects on plant health. Another obstacle is scaling the technology for large-scale agricultural use while ensuring that it remains cost-effective. Creating affordable, adaptable systems that can be integrated seamlessly with current farming practices is key to achieving widespread acceptance. Additionally, managing the energy requirements of CP generation and reducing infrastructure costs are essential to ensure the technology’s long-term economic viability.

Achieving the best results requires the careful optimization of both the process and product parameters. Key factors include the design of the CP system, the composition of the working gas, the treatment mode (direct or indirect), gas flow rate, applied voltage, exposure time, power level, relative humidity, pressure, frequency, and temperature. Addressing the challenges associated with achieving a uniform plasma distribution and increasing the treatment area are crucial for effective commercial applications. While direct CP exposure tends to be more powerful, it often lacks the uniformity provided by indirect exposure, where reactive species are distributed more consistently. Additionally, higher flow rates enhance the transport and efficiency of reactive species but may reduce treatment effectiveness due to shorter residence times. Future studies should prioritize understanding the mechanisms of CP-induced improvements in protein functionality, allergenicity reduction, and macromolecular modifications, alongside designing CP systems capable of treating larger areas with a consistent plasma distribution. CP technology offers a groundbreaking method for altering macromolecules, with potential applications ranging from the creation of highly thermally stable semiconductors to innovative food packaging materials.

## 9. Conclusions

CP technology has become a revolutionary approach for modifying proteins and lipid molecules, providing non-thermal and environmentally friendly solutions for food processing and preservation. It has been extensively shown to modify protein structures, improve functionality, and enhance lipid stability through processes such as oxidation, denaturation, and cross-linking. However, to fully harness its potential, challenges related to process standardization, scalability, and safety need to be effectively addressed. The consumer demand for raw or minimally processed foods is rising due to preferences for healthier options and increased awareness of nutrition. In recent years, CP technology has garnered considerable interest in food processing for its innovative capability to preserve agricultural products while maintaining their quality. CP enhances the microbiological safety of food without causing significant changes to its sensory, chemical, or physical properties, thus extending shelf life and ensuring superior product quality. For plant proteins, CP has shown promise in improving gelling properties; however, prolonged treatment times can lead to protein fragmentation, which may reduce their water-binding capacity. In the processing of fruits and vegetables, non-thermal plasma treatments have demonstrated benefits such as increasing drying rates, enhancing moisture diffusivity, reducing drying time and energy consumption, modifying macromolecules, and improving overall efficiency and product quality. These changes enhance nutritional value, improve functional properties, and prolong the shelf life of food products. CP has also yielded positive results in maintaining the quality and microbiological safety of various food categories. Additionally, CP treatments can inactivate enzymes by altering the secondary structure of enzyme proteins. Future research could focus on leveraging CP’s synergistic effects with other emerging technologies to create a multifaceted approach for ensuring food safety and quality, enabling broader applications in the food industry. With ongoing research advancements, CP shows significant potential as a sustainable and adaptable method for driving next-generation food innovations.

## Data Availability

Data are contained within the article.
